# Determination of Risk Factors, Incidence, and Mortality Rates of Acute Kidney Injury in COVID-19 Patients Hospitalized in the Intensive Care Unit

**DOI:** 10.3390/jcm15020483

**Published:** 2026-01-07

**Authors:** Gizem Kahraman, Pınar Karabak Bilal, Mustafa Kemal Bayar

**Affiliations:** 1 Ankara Etlik City Hospital, 06170 Ankara, Türkiye; gizemgecmisli@gmail.com; 2 Department of Anesthesiology and Reanimation, Faculty of Medicine, Ankara University, 06100 Ankara, Türkiye; mkbayar@yahoo.com

**Keywords:** COVID-19, acute kidney injury, intensive care unit, risk factors, incidence

## Abstract

**Background:** Although the main target of SARS-CoV-2 is the respiratory system, in some patients, it may affect multiple organ systems, leading to multi-organ failure. Acute kidney injury (AKI) remains one of the most frequent and clinically significant complications of severe COVID-19, with clinical importance extending beyond the acute phase due to its association with long-term renal outcomes and persistent morbidity. The incidence of AKI is particularly high among patients admitted to the intensive care unit (ICU), where its development has been consistently associated with prolonged hospitalization and increased mortality. The primary aim of this study was to determine the incidence of COVID-19-associated AKI, identify factors related to its development and severity, and evaluate mortality as a clinical outcome. **Methods:** Data from 238 COVID-19 patients monitored in the Intensive Care Unit of Ankara University Ibni Sina Hospital (ISH-ICU) between 1 January 2021 and 1 January 2022 were retrospectively reviewed. Patients were divided into two groups according to the presence of AKI. Those with AKI were staged according to KDIGO criteria (stages 1–2–3). Demographic characteristics, comorbidities, disease severity scores, laboratory parameters, and mortality outcomes were analyzed and compared between groups. **Results:** AKI was identified in 54.6% of patients. Of the patients with AKI, 32 (13.4%) had stage 1, 25 (10.5%) had stage 2, and 73 (30.7%) had stage 3 AKI. Thirteen patients (5.5%) had already developed AKI at ICU admission. AKI developed at a median of 11 days after symptom onset and 3 days after ICU admission. Advanced age, hypertension, cardiovascular disease, and chronic kidney disease were more frequent in patients with AKI (*p* < 0.001). Higher Charlson Comorbidity Index (CCI) and Acute Physiologic Assessment and Chronic Health Evaluation II (APACHE II) scores were observed in patients with stage 3 AKI. Lymphopenia and elevated levels of D-dimer, ferritin, IL-6, CRP, and procalcitonin were significantly higher in patients with stage 3 AKI than in patients with other AKI stages and the non-AKI group. Mortality rates were higher in patients with AKI and increased with advancing AKI stage (*p* < 0.001). ICU length of stay was significantly longer in the AKI group (*p* < 0.001). **Conclusions:** AKI is a common complication among critically ill patients with COVID-19 and is associated with prolonged ICU stay and higher mortality rates, particularly in advanced stages. Early identification of clinical and laboratory factors associated with AKI may support timely risk stratification and targeted management in this high-risk population.

## 1. Introduction

COVID-19 has affected millions of people, causing numerous hospitalizations, intensive care unit (ICU) admissions, and deaths [1]. Although it was initially believed that COVID-19 mainly affected the lungs, it has been found that the disease can lead to failure of multiple organs by affecting several systems, particularly the heart, gastrointestinal, and urinary systems. One of the most common and important complications of COVID-19 is AKI. Beyond its impact during the acute phase of the disease, COVID-19-related AKI has been shown to contribute to permanent renal dysfunction and long-term morbidity in survivors [2,3]. Consequently, AKI remains a primary clinical concern in COVID-19, especially among critically ill patients and in the context of post-COVID renal sequelae.

COVID-19-associated AKI has multiple risk factors and complex etiologies [4]. The pathophysiology of AKI in COVID-19 is thought to include local and systemic inflammatory and immune responses, endothelial damage and activation of coagulation pathways, and the renin–angiotensin system. However, the suggestion that the virus directly affects the renal endothelium remains controversial [5]. Advanced age, male gender, obesity, DM, hypertension, cardiovascular disease, low initial estimated glomerular filtration rate, high interleukin-6 level, mechanical ventilation, and vasopressor drug requirement are independent risk factors for AKI [6,7]. In critically ill patients with COVID-19, AKI has also been shown to be associated with increased hospital mortality and prolonged length of stay [8,9].

It has been shown that COVID-19 infection is more severe in patients who develop stage 3 AKI and is associated with prolonged hospital care or a high mortality rate [10]. The frequent occurrence of AKI requiring renal replacement therapy in COVID-19 patients monitored in the ICU significantly increases healthcare costs. Mortality rates in stage 3 AKI exceed 80% [11]. These reasons underscore the importance of identifying risk factors for the development of AKI in COVID-19 patients monitored in the ICU.

Accordingly, the primary aim of this study was to determine the incidence of AKI, identify factors associated with AKI development and severity in COVID-19 patients admitted to the ICU, and evaluate mortality as a secondary clinical outcome.

## 2. Materials and Methods

After obtaining approval from the Ankara University Faculty of Medicine Human Research Ethics Committee, dated 27 April 2022 and numbered İ05-257-22, the patients’ data were retrospectively reviewed. This study was conducted in accordance with the principles of the Declaration of Helsinki and was registered at ClinicalTrials.gov (No. NCT07089693).

### 2.1. Patients and Study Design

The data of 306 patients aged 18 years or older who were hospitalized in the Intensive Care Unit of Ankara University Ibni Sina Hospital (ICU-ISH) between 1 January 2021 and 1 January 2022 due to COVID-19 and whose COVID-19 diagnosis was confirmed by SARS-CoV-2 PCR were retrospectively analyzed. After excluding patients with end-stage renal failure, a history of kidney transplantation, those who stayed in the ICU for less than 48 h, and those with incomplete data, 238 patients were included in the study. Patients with missing essential information were excluded from the final analysis to ensure data completeness.

### 2.2. Data Collection

The diagnosis and staging of AKI were based on serum creatinine levels according to the KDIGO 2012 guidelines. We recorded the baseline serum creatinine value as the patients’ previous serum creatinine values, if any medical records from the pre-COVID-19 period existed. The serum creatinine level at admission for patients with no prior creatinine measurements was accepted as the baseline.

Patients were divided into two groups, AKI and non-AKI, according to the level of renal damage. We staged patients with AKI into subgroups (stage 1, stage 2, and stage 3) based on disease severity according to the KDIGO guidelines.

Demographic data, comorbidities and Charlson Comorbidity Index (CCI) (Table 1), symptom onset dates, hospital admission dates, number of days in hospital, Acute Physiologic Assessment and Chronic Health Evaluation II (APACHE II) scores, routine laboratory results (at admission, highest and lowest, median), respiratory support treatments received and duration, FiO_2_ values, administered antiviral and inotrope–vasopressor treatments, development and stages of AKI, and 28-day and 3-month mortality rates during ICU admission were recorded for the patients included in the study. The data were compared between groups, and factors affecting the development of AKI and mortality in patients were analyzed.

### 2.3. Statistical Analysis

Statistical analyses were performed using IBM SPSS Statistics for Windows, Version 20.0 (IBM Corp., Armonk, NY, USA). Categorical variables were compared between groups using the Pearson chi-square test or Fisher’s exact test, as appropriate, and were expressed as frequencies and percentages.

The distribution of continuous variables was assessed using a combination of graphical methods (histograms and Q–Q plots) and the Kolmogorov–Smirnov test. Given the sample size, graphical evaluation was considered the primary determinant in assessing normality. Continuous variables that did not demonstrate an approximately normal distribution were analyzed using non-parametric methods.

For comparisons between two independent groups, the Mann–Whitney U test was used for non-normally distributed variables. Comparisons across more than two groups were performed using the Kruskal–Wallis test, and post hoc subgroup analyses were conducted with Bonferroni correction when appropriate. Results for non-normally distributed variables are reported as median, interquartile range, minimum, and maximum values, along with mean and standard deviation where relevant for descriptive purposes.

Only the age variable demonstrated an approximately normal distribution based on graphical assessment and was therefore analyzed using one-way analysis of variance (ANOVA) with Tukey’s post hoc test.

To identify predictors of renal injury stages (four categories), multinomial logistic regression analysis was performed. Variables showing statistical significance at *p* < 0.05 in univariate analyses and demonstrating no significant collinearity were included in the regression model. A *p* value of <0.05 was considered statistically significant for all analyses.

## 3. Results

Upon assessment and staging of AKI in 238 patients, it was observed that 108 patients did not develop AKI (45.4%), 32 patients developed stage 1 AKI (13.4%), 25 patients developed stage 2 AKI (10.5%), and 73 patients developed stage 3 AKI (30.7%). AKI was identified in 13 patients (5.5%) before their admission to the ICU. The earliest time of AKI development in patients was 7 days before ICU admission, and the latest was 36 days after ICU admission, with the median hospitalization day for AKI development being 3 days. When the relationship between AKI and the time of COVID-19 symptom onset was examined, it was determined that AKI developed on the median 11^th^ day of symptom onset (Table 2).

Table 3 presents the demographic and laboratory data, APACHE-II and CCI scores, and mortality rates of patients based on AKI development and stage. Of the 238 patients included in the study, 105 (44%) were female, 133 (55%) were male, and the mean age of all patients was 67 ± 16 years. The mean age of the AKI group was significantly higher than that of the non-AKI group (*p* < 0.001). There was no significant difference in mean age among the stage 1, 2, and 3 AKI groups. When the sex distribution of the patients was examined according to their AKI, no significant difference was found between the AKI and non-AKI groups.

When the comorbidities of the patients were examined, the group developing AKI had significantly higher rates of hypertension (*p* = 0.032), cardiovascular disease (*p* = 0.001), and chronic kidney disease (*p* < 0.001) compared to the group not developing AKI.

After analyzing patients’ APACHE II scores, patients with stage 2 or 3 AKI had significantly higher scores than those with stage 1 AKI or without AKI (*p* < 0.001). Charlson Comorbidity Indexes for patients with stage 2 and stage 3 AKI were significantly higher than those in the non-AKI group (*p* < 0.001).

Upon examination of the patients’ laboratory data, the lowest lymphocyte counts were significantly lower in patients with stage 2 and stage 3 AKI than in patients without AKI or with stage 1 AKI (*p* < 0.001). When patients’ ferritin levels were examined, ferritin levels were significantly increased in all AKI groups compared with the non-AKI group, with the highest levels observed in stage 3 (*p* < 0.001).

The highest fibrinogen (*p* = 0.006), D-dimer (*p* = 0.002), CRP (*p* < 0.001), and procalcitonin (*p* < 0.001) levels in patients were significantly higher in those with stage 2 and 3 AKI than in patients without AKI or with stage 1 AKI. Furthermore, procalcitonin levels at the time of admission to the ICU were significantly higher in patients with stage 2 and stage 3 AKI compared to patients without AKI (*p* < 0.001). The highest serum IL-6 levels and IL-6 levels at ICU admission were significantly higher in all AKI stage groups than in patients without AKI, with the highest levels observed in the stage 3 AKI group (*p* < 0.001).

By assessing the mortality rate in patients based on the presence and stage of AKI, mortality in groups with stage 2 and stage 3 AKI was significantly higher than in groups without AKI and stage 1 AKI. Moreover, as the AKI stage increased, 28-day, 3-month, and in-hospital mortality also increased (*p* < 0.001).

Multinomial logistic regression analysis was performed to identify independent factors associated with different stages of AKI, using patients without AKI as the reference group (Table 4). In stage 1 AKI, higher baseline serum creatinine was the only variable independently associated with AKI development (OR: 34.68, 95% CI: 3.13–383.99; *p* = 0.004). For stage 2 AKI, both increasing age (OR: 1.09, 95% CI: 1.02–1.17; *p* = 0.017) and serum creatinine levels (OR: 23.75, 95% CI: 1.55–363.38; *p* = 0.023) were independently associated with AKI occurrence. In contrast, stage 3 AKI was associated with multiple indicators of severe disease, including elevated serum creatinine (OR: 170.72, 95% CI: 12.48–2335.83; *p* < 0.001), higher procalcitonin levels (OR: 2.06, 95% CI: 1.06–3.99; *p* = 0.034), lower arterial pH values (OR: 0.000, 95% CI: 0.000–0.019; *p* = 0.003), and the requirement for invasive mechanical ventilation (OR: 73.98, 95% CI: 7.51–728.68; *p* < 0.001). Other laboratory parameters, including neutrophil count, CRP, IL-6, lactate, and PaO_2_, as well as comorbid conditions and vaccination status, were not independently associated with the development of any AKI stage in the multivariable model.

Follow-up time with a nasal cannula and a simple mask was significantly longer in the non-AKI group (*p* < 0.001), and follow-up time with invasive mechanical ventilation (IMV) was significantly longer in the AKI group (*p* < 0.001). The need for inotropic vasopressors was considerably higher in the group that developed AKI (*p* < 0.001). The median length of stay in the ICU was significantly longer in the AKI group (11 days) compared to the non-AKI group (8 days) (*p* < 0.001) (Table 5).

## 4. Discussion

In this study, the frequency of AKI and the relationship between its stages and clinical and laboratory features were investigated in patients with COVID-19 requiring ICU admission. Based on the logistic regression analysis, higher baseline creatinine levels were independently associated with all stages of AKI, while advanced age was a significant predictor of stage 2 AKI. Additionally, the need for invasive mechanical ventilation, the presence of metabolic acidosis, and high procalcitonin levels were independently associated with the development of stage 3 AKI. Our findings indicate that AKI severity in critically ill COVID-19 patients is associated with systemic inflammation, respiratory failure, and disease severity.

In the literature, the incidence of AKI in COVID-19 patients followed in the ICU has been reported to range from 19.1% to 80% [7,12,13]. In our study, this rate was 54.6%. In different studies conducted in the United Kingdom and Norway, AKI was detected in 36.1% and 32% of COVID-19 patients admitted to the ICU, respectively, at the time of admission [14,15]. In the present study, AKI was detected in only 5.5% of patients at the time of admission to the ICU. The heterogeneity in AKI incidence can be attributed to differences in patient profiles across centers, length of hospital stay, medical care services, frequency of renal function monitoring, and ICU admission criteria [16,17].

In the United States, Hirsch et al. studied 5449 COVID-19 patients and demonstrated a significant association between AKI and advanced age, invasive mechanical ventilation, and vasopressor requirement [6]. Sabaghian et al. [18] reported that the most common comorbidities in patients who developed AKI were diabetes, hypertension, hyperlipidemia, and chronic kidney disease. Similarly, Bajgain et al. [19] reported that hypertension, diabetes, and cardiovascular diseases were common comorbidities in COVID-19 patients and were associated with higher mortality rates. In our study, advanced age and specific comorbidities (particularly hypertension, cardiovascular diseases, and chronic kidney disease) were associated with AKI development, and 96.3% of deceased patients had at least one comorbidity. Advanced age and the presence of comorbidities were also significantly associated with mortality. Moreover, the association between stage 3 AKI and higher CCI and APACHE II scores observed in our study is consistent with the literature [20].

In our cohort, more advanced AKI stages were associated with marked lymphopenia and significantly higher levels of inflammatory and coagulation markers, including D-dimer, ferritin, CRP, procalcitonin, and IL-6. These findings are in line with earlier ICU-based COVID-19 cohorts and may reflect the coexistence of endothelial injury, hypercoagulation, and systemic inflammation in patients with COVID-19-related kidney injury [18,21,22,23,24,25].

Previous studies have demonstrated that AKI in critically ill COVID-19 patients is associated with increased ICU mortality and prolonged length of stay [26,27]. Similarly, in our study, patients who developed AKI had more extended hospital and ICU stays and higher 28-day and 3-month mortality rates, with the highest mortality observed in stage 3 AKI. These findings suggest that AKI severity is closely associated with adverse outcomes in COVID-19 patients and underscore the importance of early identification and close monitoring of patients at high risk for AKI.

### Study Limitations

This study has several limitations. Firstly, the single-center, retrospective design may limit generalizability, and data obtained from medical records may contain errors. Secondly, the lack of detailed data, including exposure to nephrotoxic drugs, hemodynamic changes, urine output, urinalysis findings, and advanced diagnostic assessments, prevented precise classification of AKI etiology. Thirdly, mortality was assessed as all-cause mortality; therefore, the influence of unmeasured factors cannot be completely ruled out. Finally, viral variants and treatment strategies may have changed during the study period, and data on those variables are not available in our study. Fourthly, although mortality was a key clinical outcome, a separate multivariable model for mortality was not constructed, as the primary aim of this study was to identify factors associated with AKI development and severity. Therefore, mortality findings should be interpreted in the context of AKI stage and overall disease severity.

Despite these limitations, our findings provide essential information regarding AKI risk factors and outcomes in critically ill patients with COVID-19.

## 5. Conclusions

AKI is a common and clinically significant complication in critically ill COVID-19 patients. The severity of AKI is closely associated with prolonged ICU stay and increased mortality. Therefore, early diagnosis and timely intervention, including preventive and supportive measures, are vital for patients at high risk of AKI.

## Figures and Tables

**Table 1:** The Charlson Comorbidity Index (CCI).

Condition	Points
Age ≤ 40	0
40 < Age ≤ 50	1
50 < Age ≤ 60	2
60 < Age ≤ 70	3
Age > 70	4
Myocardial infarction	1
Congestive heart failure	1
Peripheral vascular disease	1
Cerebrovascular disease	1
Dementia	1
Chronic lung disease	1
Connective tissue disease	1
Moderate liver disease	1
Diabetes mellitus without end-organ damage	1
Diabetes mellitus with end-organ damage	2
Hemiplegia	2
Kidney disease	2
Any malignancy	2
Lymphoma	2
Leukemia	2
Severe liver disease	3
Metastatic solid tumor	6
AIDS	6
Peptic ulcer	1

**Table 2:** The development and timing of acute kidney injury (AKI) in the study population.

AKI stage, *n* (%)	
No AKI	108 (45.4)
Stage 1	32 (13.4)
Stage 2	25 (10.5)
Stage 3	73 (30.7)
AKI present at ICU admission, *n* (%)	13 (5.5)
Time to AKI development during ICU stay, days (median, min–max)	3 (−7 to 36)
Time from symptom onset to AKI development, days (median, min–max)	11 (0–44)

AKI: acute kidney injury; ICU: intensive care unit. Negative values indicate AKI occurrence before ICU admission.

**Table 3:** The general characteristics of patients according to AKI development and stage.

	No AKI	Stage 1 AKI	Stage 2 AKI	Stage 3 AKI	*p* Value
Age, years (mean ± SD)	61 ± 17	70 ± 15	74 ± 16	72 ± 14	<0.001
Sex, *n* (%)					
Female	50 (47.6)	13 (12.4)	8 (7.6)	34 (32.4)	0.437
Male	58 (43.6)	19 (14.3)	17 (12.8)	39 (29.3)	0.437
Comorbidities, *n* (%)					
Hypertension	14 (10.2)	20 (14.6)	52 (38)	51 (37.2)	0.032
Diabetes mellitus	30 (41.7)	10 (13.9)	7 (9.7)	25 (34.7)	0.819
Cardiovascular disease	16 (18.4)	15 (17.2)	26 (29.9)	30 (34.5)	0.001
Cerebrovascular disease	7 (35.0)	2 (10.0)	3 (15.0)	8 (40.0)	0.626
Neurological disease	18 (45.0)	3 (7.5)	5 (12.5)	14 (35.0)	0.628
Malignancy	19 (37.3)	9 (17.6)	3 (5.9)	20 (39.2)	0.198
Chronic lung disease	25 (41.7)	10 (16.7)	9 (15.0)	16 (26.7)	0.419
Chronic kidney disease	9 (15.5)	15 (25.9)	8 (13.8)	26 (44.8)	<0.001
Chronic liver disease	4 (40.0)	1 (10.0)	1 (10.0)	4 (40.0)	0.928
Immunosuppression	18 (48.6)	3 (8.1)	4 (10.8)	12 (32.4)	0.782
Severity scores (mean ± SD)					
APACHE II	16 ± 10	16 ± 11	21 ± 11	27 ± 10	<0.001
CCI	3 ± 3	5 ± 3	5 ± 3	6 ± 3	<0.001
Laboratory parameters (median)					
Lymphocyte (×10^9^/L)—admission	0.65	0.68	0.64	0.54	0.156
Lymphocyte (×10^9^/L)—lowest	0.50	0.42	0.27	0.29	<0.001
Ferritin (ng/mL)—admission	586.6	685.1	543.6	688.6	0.360
Ferritin (ng/mL)—highest	765.7	12,355	10,380	32,650	<0.001
Fibrinogen (g/L)—admission	5.64	4.92	6.29	5.15	0.318
Fibrinogen (g/L)—highest	5.64	6.07	7.27	6.63	0.006
D-dimer (ng/mL)—admission	541	881	545	916	<0.093
D-dimer (ng/mL)—highest	1953	1236.5	2953	3622	0.002
CRP (mg/L)—admission	94.7	80.3	158.0	108.0	0.014
CRP (mg/L)—highest	114.9	103.3	248.7	215.0	<0.001
Procalcitonin (ng/mL)—admission	2.25	2.77	3.81	4.20	<0.001
Procalcitonin (ng/mL)—highest	3.19	2.15	5.13	7.36	<0.001
IL-6 (pg/mL)—admission	26.84	31.76	46.83	75.13	<0.001
IL-6 (pg/mL)—highest	47.92	66.93	74.03	133.3	<0.001
Mortality, *n* (%)					
In-hospital mortality	18 (16.7)	10 (31.2)	16 (64.0)	66 (90.4)	<0.001
28-day mortality	18 (16.7)	10 (31.2)	14 (56.0)	56 (76.7)	<0.001
3-month mortality	26 (24.1)	10 (31.2)	19 (76.0)	66 (90.4)	<0.001

AKI was classified according to KDIGO criteria. AKI: acute kidney injury; CCI: Charlson Comorbidity Index; CRP: C-reactive protein; IL-6: interleukin-6.

**Table 4:** Multinomial logistic regression analysis of factors associated with AKI stages.

	Stage 1 AKI OR (95% CI)	*p* Value	Stage 2 AKI OR (95% CI)	*p* Value	Stage 3 AKI OR (95% CI)	*p* Value
Age (years)	1.03 (0.99–1.08)	0.164	1.09 (1.02–1.17)	0.017	1.03 (0.96–1.10)	0.461
Serum creatinine	34.68 (3.13–383.99)	0.004	23.75 (1.55–363.38)	0.023	170.72 (12.48–2335.83)	<0.001
Procalcitonin	1.19 (0.65–2.19)	0.568	1.63 (0.85–3.13)	0.143	2.06 (1.06–3.99)	0.034
Arterial pH	0.32 (0.00–367.75)	0.751	0.004 (0.00–16.55)	0.193	0.000 (0.000–0.019)	0.003
Invasive mechanical ventilation	2.00 (0.56–7.16)	0.288	2.58 (0.41–16.18)	0.312	73.98 (7.51–728.68)	<0.001

Multinomial logistic regression analysis was performed to identify independent factors associated with different stages of acute kidney injury (AKI), using patients without AKI as the reference group. Odds ratios (ORs) are presented with 95% confidence intervals (CIs). Variables included in the model were selected based on clinical relevance and previous literature. Only variables independently associated with AKI stages in the multivariable model are presented for clarity.

**Table 5:** The effects of respiratory therapy, ICU length of stay, and inotropic administration on outcomes in AKI and non-AKI groups.

	Non-AKI (*n* = 108)	AKI (*n* = 130)	*p* Value
Respiratory support and day, median (min–max)			
Nasal cannula or simple mask (days)	3 (0–13)	1 (0–11)	*p* < 0.001
Reservoir mask (days)	0 (0–3)	0 (0–11)	*p* = 0.571
HFNO or NIV (days)	2 (0–18)	3 (0–19)	*p* = 0.017
IMV (days)	0 (0–29)	7 (0–74)	*p* < 0.001
Inotrope–vasopressor requirement, *n* (%)	25 (23.1)	98 (75.4)	*p* < 0.001
ICU length of stay (days), median (min–max)	8 (2–35)	11 (2–85)	*p* < 0.001

AKI: acute kidney injury; ICU: intensive care unit; HFNO: high-flow nasal oxygen; NIV: non-invasive ventilation; IMV: invasive mechanical ventilation.

## Data Availability

The data presented in this study are available upon request from the corresponding author.
